# Bilingualism, Executive Function, and the Brain: Implications for Autism

**DOI:** 10.1162/nol_a_00057

**Published:** 2021-12-23

**Authors:** Celia Romero, Lucina Q. Uddin

**Affiliations:** Department of Psychology, University of Miami, Coral Gables, FL, USA; Neuroscience Program, University of Miami Miller School of Medicine, Miami, FL, USA; Department of Psychiatry and Biobehavioral Sciences, University of California Los Angeles, Los Angeles, CA, USA

**Keywords:** bilingualism, executive function, autism spectrum disorder, cognition, second language exposure, dual language

## Abstract

*Autism spectrum disorder* (ASD) is associated with marked heterogeneity with respect to the development of executive function abilities. The *bilingual advantage* refers to the observation that individuals who speak two languages perform better on executive function tasks than monolinguals under some circumstances. There is not yet consensus, however, as to whether this advantage can be reliably demonstrated, nor is there consensus regarding under which conditions it emerges. Bilingual and monolingual children with ASD have comparable developmental outcomes, particularly in the areas of core ASD symptoms, cognitive function, and language. Still, despite the potential advantages that bilingualism may confer, clinicians commonly advise against providing a bilingual environment for children with ASD. The purpose of the present review is to provide an up-to-date assessment of the limited literature on bilingualism in children with ASD in order to inform evidence-based practice. Studies suggest a potential bilingual advantage in ASD in the areas of nonverbal intelligence quotient, adaptive functioning, and expressive vocabulary. A limited yet growing literature provides preliminary evidence for enhanced executive function ability in some children with ASD. Taken together, current evidence suggests that although a bilingual advantage may not be universally present in typical development, it may manifest under specific circumstances, conferring advantage for populations in which executive function is compromised. Further work is needed to develop consistent, evidence-based guidelines around language recommendations for families of children with ASD and to better understand the cognitive and brain mechanisms giving rise to the bilingual advantage in clinical developmental populations.

## INTRODUCTION

Approximately 1 in 54 children in the United States are diagnosed with autism spectrum disorder (ASD; Maenner et al., 2020). ASD is associated with marked heterogeneity with respect to the development of executive function abilities including planning for future goals, inhibiting maladaptive responses, maintaining and manipulating information in working memory, and flexibly adapting to changes in the environment (Baez et al., 2020; Banich, 2009). While over 20% of children in the United States speak a language other than English at home, and approximately 12 million American children are raised bilingually (Annie E. Casey Foundation, 2018), there remains a lack of evidence to guide clinical practice for parents of children with ASD regarding language use. This is in part due to unresolved issues with respect to the cognitive and neural mechanisms supporting the development of bilingualism.

The bilingual experience is thought to have influences beyond language processing; however, it was initially assumed that these consequences were negative. Despite past assumptions that learning multiple languages may cause confusion and disruptions in language development, current findings demonstrate no differences in language acquisition, proficiency, and achievement of milestones between monolingual and bilingual typically developing children (Dai et al., 2018; Hambly & Fombonne, 2012; Petersen et al., 2012; Valicenti-McDermott et al., 2013). Furthermore, emerging evidence suggests that bilingualism may be associated with cognitive advantages, particularly within the executive function domain (Gonzalez-Barrero & Nadig, 2019; Iarocci et al., 2017; Ratto et al., 2020; Sharaan et al., 2021).

Recent research has produced mixed results, with some studies reporting a *bilingual advantage* and others failing to find significant effects in typically developing children and neurotypical adults (Dick et al., 2019; Nichols et al., 2020), and some even reporting a bilingual disadvantage (Bialystok & Shapero, 2005). It is still unclear whether or not a bilingual advantage for executive function truly exists, and under what conditions it manifests. Nonetheless, current recommendations for typically developing children are to encourage and support bilingual exposure at home (US Departments of Health & Human Services & Education, 2016).

Despite the potential advantages that bilingualism may confer with regards to executive function abilities, mixed and limited research exists specific to bilingualism in children with ASD. This dearth of information leaves clinicians struggling to develop informed recommendations for families of children with this increasingly prevalent neurodevelopmental condition. The current review aims to summarize what is known about the influence of bilingual exposure on executive function development and related cognitive outcomes in autism in order to aid clinicians and parents of children with ASD regarding their decision on how many languages to speak in the home to best support cognitive development. In addition, we aim to provide a brief synopsis of current views on how bilingualism impacts the neural basis of executive function systems in typical and atypical development.

## EXECUTIVE FUNCTION

Research investigating the impact of bilingualism on cognitive development has mainly focused on executive function. *Executive function* is an umbrella term used to describe a set of cognitive processes that govern goal-directed behavior. Generally, executive function encompasses neurocognitive processes including cognitive flexibility, inhibitory control, and working memory (Blair, 2016; Diamond, 2013; Garon et al., 2008; McClelland & Cameron, 2012; Miyake et al., 2000). These processes are necessary for flexible problem-solving, selective attention, ignoring distractions, and retention of information, and ultimately depend on the integrity of neural networks involving lateral frontoparietal (L-FPN), midcingulo-insular (M-CIN), and frontostriatal brain networks (Dajani & Uddin, 2015; Miller & Cohen, 2001; Seeley et al., 2007; Uddin, 2021a; Zelazo & Lee, 2010). The M-CIN is often referred to as the salience network or the cingulo-opercular network and includes the bilateral anterior insulae, the anterior midcingulate cortex, and subcortical nodes (Uddin, 2021a; Uddin et al., 2019). The L-FPN is also known as the *executive control network* and includes lateral prefrontal cortices (PFCs), ventrolateral PFC and inferior frontal junction, the inferior parietal lobule, posterior inferior temporal lobes, and parts of the midcingulate gyrus (Uddin, 2021a).

Across development, executive function enables individuals to perform increasingly complex tasks, and distinctive aspects of executive function reflect different periods of growth (for an overview of executive function development, see Best & Miller, 2010; Garon et al., 2008). During infancy and preschool, core components of executive function develop and form the foundation for the development of higher cognitive processes well into adulthood (Garon et al., 2008).

### Executive Function in Children With ASD

Executive function skills have been demonstrated to be impaired in children with ASD within laboratory settings and in daily life activities (Demetriou et al., 2018; C. L. E. Lai et al., 2017); however, the profile of executive function in autism is characterized by considerable heterogeneity (Baez et al., 2020; Dajani et al., 2016; Geurts et al., 2014). A recent meta-analysis demonstrates relatively stable broad executive dysfunctions in ASD (Demetriou et al., 2018) that have been proposed to make significant contributions to core deficits in ASD, particularly restricted and repetitive behaviors (RRBs; Mostert-Kerckhoffs et al., 2015). RRBs are considered hallmark features of ASD and include stereotyped movements, insistence on sameness, and restricted interests (American Psychiatric Association, 2013). RRB severity is related to cognitive inflexibility in children with ASD (Lopez et al., 2005). Research investigating the neural circuitry underlying RRBs highlights the role of frontostriatal systems in mediating these behaviors (Wilkes & Lewis, 2018).

There is evidence to suggest specific executive function deficits in cognitive flexibility among individuals with ASD (Baez et al., 2020; Yerys et al., 2015; Yeung et al., 2016), particularly in younger children (Schmitz et al., 2006; van den Bergh et al., 2014). In ASD, brain abnormalities have been observed in cortical volume and thickness in frontal and other cortical brain regions (Frazier & Hardan, 2009; Kemper & Bauman, 1998; Romero-Garcia et al., 2019; Turner et al., 2016). Atypical brain responses in individuals with ASD while performing executive function tasks have been extensively documented (Braden et al., 2017; Garic et al., 2019; Hanaie et al., 2018; Just et al., 2007; Walsh et al., 2019). A recent review of neural mechanisms underpinning cognitive inflexibility in autism finds atypical patterns of L-FPN and M-CIN activation during set-shifting and task-switching executive function tasks (Uddin, 2021b).

The Executive Dysfunction model of ASD proposes a domain-general deficit in executive functions that widely affects ASD symptoms (Hill, 2004; Pennington & Ozonoff, 1996). The model originally implied a relationship between cognitive inflexibility and preservation in ASD; however, to date research has found a wider influence of executive function in ASD symptomatology, including mental health, disability, social cognition, and quality of life (May & Kana, 2020). A recent meta-analysis across many brain imaging studies of executive functions in individuals with ASD found different patterns of frontoparietal activation during executive function tasks (May & Kana, 2020). The findings highlight neurobiological variations in executive function network recruitment that may contribute to the development of an executive dysfunction profile ASD. These results support the executive dysfunction hypothesis of ASD and suggest that deficient frontoparietal recruitment may underlie executive function difficulties experienced by individuals with ASD. Individual differences in executive function profiles between children with ASD are evident; however, the factors influencing executive function development and outcomes in ASD are not fully understood (Demetriou et al., 2018). Bilingual exposure, currently understudied, may be one of these factors.

## PROPOSED MECHANISMS UNDERLYING THE EXECUTIVE FUNCTION BILINGUAL ADVANTAGE

The concept of a *bilingual advantage* suggests that individuals fluent in two languages develop cognitive advantages in the executive function domain. There is no clear consensus about the specific cognitive and neural mechanisms underlying the proposed bilingual advantage in executive function; however, it is hypothesized to be based on neuroplasticity, or the brain’s adaptivity and formative ability gained via experience throughout one’s life (Bialystok, 2009, 2017). Inhibition and monitoring are posited to be potential cognitive mechanisms conferring enhanced executive control in individuals with diverse language experiences (Bialystok, 2017; Bialystok et al., 2012). This model contends that both languages in a bilingual individual’s repertoire are always active, and there is thus a continual competition for selection. Joint activation and language switching are significant aspects of bilingual language control implicating nonverbal cognitive processes in their engagement. In order to manage and resolve competition between the two languages, bilinguals deploy a network of frontoparietal brain regions more generally involved in action selection and cognitive control (Carlson & Meltzoff, 2008). Lifelong experience managing two competing languages imposes demands on the cognitive system that are not typically utilized for language processing. Subsequently, the brain adapts to these recurrent demands and reorganizes networks to build more efficient executive control mechanisms, resulting in cognitive benefits when non-linguistic processing draws on the same executive control networks (Rodríguez-Pujadas et al., 2013). Since language switching involves overlapping frontal brain systems involved in executive control and inhibitory processes (Coderre et al., 2016), it is proposed that bilingualism may result in the general enhancement of these control systems in the brain (Bialystok et al., 2012; Hilchey & Klein, 2011). Neural adaptation may occur via change in structural resources or capacity, alterations in regional efficiency, or fluctuations in network connectivity (Bialystok, 2017).

Adaptations to language control demands in bilinguals are evident in the structural plasticity of the brain (Burgaleta et al., 2016; Mamiya et al., 2016; Mechelli et al., 2004) and neural activity modulation (Abutalebi et al., 2012; Anderson et al., 2018; Ansaldo et al., 2015; Bialystok et al., 2005; Dash et al., 2019; Kousaie & Phillips, 2017; Luk et al., 2010; Morales et al., 2015; Timmer et al., 2017). However, although current research suggests changes in the bilingual brain’s architecture, the nature and location of the adaptation and brain recruitment vary between studies (for discussion see García-Pentón et al., 2016).

The adaptive control hypothesis argues that language context is a key determinant of the impact of bilingualism on executive function and describes three types of interactional language contexts requiring differing levels of inhibitory control to enable language: single-language contexts, dual-language contexts, and dense code-switching contexts (Green & Abutalebi, 2013). The contexts differ in the extent of demand in language control based on the presence or absence of both languages and how language interference is resolved. For example, in single-language contexts each language is generally kept apart and is used in separate distinct environments with infrequent language switching. In a dual language context, both languages are spoken within the same environment, resulting in frequent switching. In this context, the need for language control is argued to be high because the production of both languages is kept separate (Green, 2018; Green & Wei, 2014). In a dense code-switching language context, languages are interchanged within single statements, or words are adapted from one language to integrate with the other language. The adaptive control hypothesis identifies eight control mechanisms engaged in the differing bilingual contexts: goal maintenance, conflict monitoring, interference suppression, salient cue detection, selective response inhibition, task engagement, task disengagement, and opportunistic planning (Green & Abutalebi, 2013). Central to this framework is that similarly to general cognitive control, bilingual language control fluctuates depending on the environment’s linguistic control demands. Thus, the extent to which each interactional language context requires inhibitory control will determine the magnitude of the bilingual advantage. However, recent research in English-Mandarin young adults has found positive and moderately strong correlations between engagement in dual-language context and dense code-switching context, suggesting fluidity between engagement in the three language contexts (G. Lai & O’Brien, 2020). Therefore, the model’s distinction of interactional language contexts may be less pronounced in multilingual environments characterized by prevalent bilingualism and wide use of multiple languages.

### Bilingual Executive Function Advantage in the General Population

Executive function is generally assessed with self- or informant-report questionnaires or laboratory task-based measures. Studies assessing the relationship between bilingualism and executive function abilities generally utilize a between-subject design and examine the performance of bilingual and monolingual individuals on tasks including one condition that demands an aspect of executive function. Classic tasks frequently utilized in these studies include the dimensional change card sort (Zelazo, 2006), the flanker task (Eriksen & Eriksen, 1974), the Simon task (Simon & Rudell, 1967), the color-shape switching task (Prior & MacWhinney, 2010), and the attention network test (Rueda et al., 2004).

Of all executive function processes, inhibition has been most widely studied. The flanker and Stroop tasks are thought to measure inhibitory control by inducing cognitive conflict in slightly different manners. In the Simon task, colored squares are used to induce conflict by a spatial-stimulus-response mismatch in incongruent trials, whereas, in the flanker task, congruent or incongruent arrays of arrows are utilized to measure resistance to the interference of flanking distractors. In these tasks, beyond inspecting overall reaction times in the congruent and incongruent trials, a difference score as an index of inhibitory control is calculated. Comparisons of task performance among monolingual and bilingual children demonstrate smaller costs (Mezzacappa, 2004; Yoshida et al., 2011) or more accurate and faster performance (Yang et al., 2011) for bilingual children on the incongruent trials. Evidence suggesting bilingualism enhances aspects of executive function on tasks that require ignoring relevant information, task switching, and resolving conflict has been championed and replicated many times (Barac et al., 2014; Bialystok et al., 2012; Costa et al., 2009; Hartanto & Yang, 2020; Kroll & Bialystok, 2013; Prior & MacWhinney, 2010). However, there is no consensus as yet on the casual relationship between bilingualism and these benefits and whether these benefits constitute an advantage in real life.

Over the past two decades, numerous studies have tested the bilingual advantage but no consensus has yet been reached (for meta-analyses and reviews see Donnelly et al., 2019; Paap, 2019; van den Noort et al., 2019). Bilingualism has been demonstrated to have a positive effect on executive function in children (Carlson & Meltzoff, 2008; Crivello et al., 2016; Kempert & Hardy, 2015; Martin-Rhee & Bialystok, 2008); yet, there is a gap in our understanding of the effects of bilingualism on cognitive development outside of the language domain in both typically developing children and children with neurodevelopmental conditions.

More recently, studies have continued to produce mixed results, with some studies reporting a bilingual advantage (Hartanto & Yang, 2020) and others failing to replicate these results in neurotypical adults and typically developing children. Current large-sample studies of neurotypical adults and typically developing children do not support the bilingual advantage hypothesis. A large-sample study (*n* = 4,524) assessing bilingualism in 9–10-year-old typically developing children does not support the bilingual advantage hypothesis for inhibitory control, attention switching, or cognitive flexibility (Dick et al., 2019). Similarly, Nichols and colleagues (2020) find no reliable executive function differences between language groups in 11,041 adults. A recent meta-analysis by Gunnerud et al. (2020) offers limited support for a bilingual executive function advantage in 2–14-year-old typically developing children. Moderator analysis conducted by the experimenters shows substantial heterogeneity of true effects and unexplained heterogeneity in the effect sizes.

Research investigating bilingualism and cognition using functional magnetic resonance imaging (fMRI) methods in typical populations have also resulted in mixed findings. Limited existing neuroimaging studies suggesting a bilingual advantage are underpowered, report statistically nonsignificant differences in executive function task performance across monolingual and bilingual groups, and fail to directly relate brain activation with behavioral performance (Costa & Sebastián-Gallés, 2014; Garbin et al., 2010; Mohades et al., 2014). More recently, DeLuca et al. (2020) suggest that equivalent task performance among language exposure groups allows for meaningful interpretation of functional neural differences without possible confounds of behavioral differences. However, interpreting bilingualism-induced brain differences merely from neural activation patterns in the absence of statistically different task-performance effects may lead to the reverse inference fallacy (Poldrack, 2006).

The presence of a bilingual advantage in non-linguistic processes, particularly within the executive function domain, have been brought into question by many studies in which differences between bilinguals and monolinguals cannot be consistently replicated. Literature on bilingualism and executive function is proposed to be affected by a confirmation bias to report only positive results (de Bruin et al., 2015; Paap & Greenberg, 2013). Findings suggesting a bilingual advantage have been debated due to replication failures and claims that they are simply artefacts of small non-representative sample studies that do not adequately control for potential confounds (Lehtonen et al., 2018; Paap & Greenberg, 2013; von Bastian et al., 2016). Definitions of bilingualism differing between authors and fields also contribute to the conflicting results found in bilingualism research (Luk & Bialystok, 2013). Similarly, varying bilingualism parameters including the number of languages known (Schroeder & Marian, 2016), age of acquisition of each language (Johnson & Newport, 1989), proficiency in each language (Perani, 1998), or language-switching habits (Verreyt et al., 2015) influence distinct neurocognitive processes.

Nonetheless, current research investigating a potential bilingual advantage among neurotypical populations aligns with the conclusion drawn from Paap et al.’s (2015) narrative review suggesting that there is either no bilingual advantage in typically developing children and neurotypical adults, or it is “restricted to very specific and undetermined circumstances” (Paap et al., 2015 title).

### Bilingual Executive Function Advantage May Exist Under Certain Conditions

The extent to which bilingualism confers advantages in the executive function domain in the general population is still an ongoing topic of debate and warrants further research. Conflicting findings in the literature have led researchers to argue whether the bilingual executive function advantage might be restricted to specific groups of individuals or contexts (Bak, 2016; Bialystok, 2016; de Bruin, 2019). The most robust bilingual advantage claims come from studies of individuals with executive dysfunction.

Older adults display reduced efficiency of lateral prefrontal control regions and counteract for age-related declines in executive function task performance by relying on enhanced frontotemporal connectivity compared with younger adults (Hakun et al., 2015). The default-executive coupling hypothesis of aging theorizes that inflexible coupling of the M-FPN and lateral prefrontal regions underlie worsening performance on executive function tasks and reduced flexibility (Spreng & Turner, 2019). It has been proposed that while the effects of bilingualism may not be present in the general population, lifelong experience managing multiple languages may confer an advantage later in life when executive functions are compromised.

In individuals experiencing age-related cognitive decline, a *cognitive reserve* has been observed, where the bilingual brain is more resistant to neurodegeneration and dementia (Schweizer et al., 2012). Approximately 60% of studies in the field demonstrate that lifelong use of two languages may impart advantages in the executive function domain or delay the appearance of symptoms associated with cognitive decline and dementia (Calvo et al., 2016). More recently, a study examining bilingualism using a continuum from passive to active bilingualism, identifies a certain degree of bilingualism that has neuroprotective benefits (Calabria et al., 2020). The fact that bilingual experience helps offset age-related losses in executive processes has led to the proposal that bilingualism may act as a neuroprotective factor against dementia by buffering against the decline in cognitive control abilities typically observed in later life (Costumero et al., 2020; Dash et al., 2019). Despite equal cognitive decline in monolingual and bilingual groups, studies have found increased brain atrophy in areas associated with dementia among bilingual individuals, suggesting bilinguals have a higher threshold for reaching a dementia diagnosis (Duncan et al., 2018; Schweizer et al., 2012). Other studies suggesting alternative networks where circuits not affected by neurodegeneration play a compensatory role, demonstrate greater frontoparietal network connectivity (Perani et al., 2017) and improved functional and neural efficiency within the executive control network in bilingual older adults (Abutalebi et al., 2014; Borsa et al., 2018; Gold et al., 2013; Del Maschio et al., 2018).

Similar robust bilingual advantage claims come from studies of children of lower socioeconomic status (SES; Carlson & Meltzoff, 2008; Engel de Abreu et al., 2012; Hartanto et al., 2019). It is well known that SES affects language development and modulates the development of executive function skills (Bradley & Corwyn, 2002; Lawson et al., 2018; Mezzacappa, 2004; Noble et al., 2007). Children of lower SES backgrounds perform more poorly on executive function tasks across development (see Hackman et al., 2015, for a discussion). Behavioral assessment of SES-related executive function differences is bolstered by neuroimaging evidence revealing differences in brain function and structure associated with executive function abilities among high and low SES children (Finn et al., 2017; Kishiyama et al., 2009; Noble et al., 2012; Sheridan et al., 2012, 2017).

Precocious executive function development in bilingual children may help offset SES disadvantages (Engel de Abreu et al., 2012; Kempert et al., 2011; Poarch & Bialystok, 2017). Carlson and Meltzoff (2008) demonstrated that 5–7-year-old bilingual children of lower SES backgrounds exhibited advantages over monolingual children of higher SES backgrounds on an assortment of executive function tasks (e.g., Simon says, visually cued recall, advanced dimensional change card sort) controlling for parental education as proxy for SES. While a bilingual advantage emerged only after controlling for SES, the composite raw scores of the executive function tasks revealed similar performance between lower SES bilingual children and higher SES monolingual children. These findings suggest that some of the cognitive disadvantages associated with lower SES may be compensated by superior cognitive control mechanisms resulting from bilingual experience. More recently, Naeem et al. (2018) determined that bilingualism is crucial in promoting speed of processing advantages among economically disadvantaged 18–30-year-old adults, but had little impact on individuals with high SES. Thus, bilingualism is hypothesized to protect against cognitive effects associated with poverty. It is important to note, however, that a recent meta-analysis found significant effects for 2–14-year-old middle-class SES bilinguals, while those with a low and upper-middle SES exhibited no bilingual advantage (Gunnerud et al., 2020). However, although their overall mean effect size was relatively small, true variation was identified between studies, suggesting that a bilingual advantage might be present under specific conditions. Taken together, research among populations with executive dysfunction (e.g., children of low SES backgrounds and older adults) suggests that the bilingual advantage may manifest under these and perhaps other specific circumstances.

## BILINGUALISM IN CHILDREN WITH ASD

Bilingualism in children with ASD is understudied. Currently, there are no specific guidelines for bilingual families of children with ASD, a neurodevelopmental condition characterized by difficulties in social and communication skills (Lord et al., 2018). Autism is associated with marked heterogeneity with respect to the development of spoken language. While some children do not develop any spoken language, some have a restricted range of verbal communication skills, others experience only subtle differences, and some have superior linguistic skills (Watson & Flippin, 2008).

Children with ASD also exhibit considerable heterogeneity in executive function abilities (Baez et al., 2020). This may present complications when learning verbal referents for related ideas in multiple languages. Theoretically, these executive function difficulties could present difficulties for children with ASD attempting to learn verbal referents for similar concepts in multiple languages. Thus, many parents (Hampton et al., 2017) and practitioners (Moore & Pérez-Méndez, 2006) have concerns about raising children with ASD speaking more than one language. As a result, parents of children with ASD in the United States are commonly advised to speak only one language with their children (Baker, 2013; Bird et al., 2012; Harlin & Paneque, 2006; Yu, 2013, 2016a). However, there is growing evidence that there are no negative effects of bilingualism on language comprehension, production, reading, or writing in children with ASD (Bird et al., 2012; Zhou et al., 2019).

There is very little research specific to bilingualism in children with ASD, leaving clinicians struggling to develop informed recommendations for families of children with the condition. Research on the effects of raising a child with ASD in a bilingual environment is emerging; still, the majority of studies have focused on examining only language outcomes. Current literature demonstrates that infants with ASD, even those with intellectual disability, can acquire second language vocabulary (Hambly & Fombonne, 2014; Wang et al., 2018). Current literature also demonstrates that at school-age, 6–9-year-old children with ASD can become proficient bilinguals and follow similar language development patterns as typically developing bilingual children (Gonzalez-Barrero & Nadig, 2019).

A small but growing body of evidence shows that children with ASD raised in bilingual households have equivalent or better language outcomes than children with ASD from monolingual home environments. For instance, there is similar performance in early language milestones between bilingual and monolingual toddlers with ASD (Hambly & Fombonne, 2012; Valicenti-McDermott et al., 2013). However, compared with toddlers with ASD less than 3-years-old reared in monolingual homes, bilingually exposed toddlers with ASD are found to more frequently use gestures and vocalize, with no other differences in language skills between the groups (Valicenti-McDermott et al., 2013). Researchers have also demonstrated that bilingually exposed children with ASD have receptive and expressive language abilities comparable to monolingual children with ASD (Dai et al., 2018; Petersen et al., 2012). However, 3–6-year-old bilingual children with ASD reportedly have larger total production vocabularies than children raised monolingually (Petersen et al., 2012). Together, current research suggests bilingualism has no detrimental impact on language outcomes for children with ASD (Bird et al., 2012; Gonzalez-Barrero & Nadig, 2019; Hambly & Fombonne, 2012; Ohashi et al., 2012; Reetzke et al., 2015; Wang et al., 2018).

Emerging evidence also suggests that children with ASD reared in bilingual households demonstrate developmental outcomes equivalent to or superior to children with ASD raised in monolingual homes. For instance, 3–6-year-old children exposed to bilingual environments from birth demonstrate better parent-reported social interaction skills on the Vineland Adaptive Behavior Scales-II interpersonal subdomain than children of the same age exposed after the age of 3 years (Hambly & Fombonne, 2012). Furthermore, compared with their monolingual peers, bilingual toddlers with ASD do not experience any negative effects in the social domain (Hambly & Fombonne, 2012; Reetzke et al., 2015) and demonstrate higher adaptive functioning (Valicenti-McDermott et al., 2013). In terms of symptom severity, the literature suggests that bilingual children with ASD do not experience any differences in core autism symptom severity scores as assessed with the Autism Diagnostic Observation Scale, a standardized diagnostic test for autism (Bird et al., 2012; Hambly & Fombonne, 2012; Ohashi et al., 2012; Valicenti-McDermott et al., 2013).

### Bilingual Executive Function Advantage in Children With ASD

The extent to which bilingualism is associated with enhanced executive function abilities in developmental clinical populations is debated. Bilingual research in the executive function domain among individuals with ASD remains sparse. Iarocci et al. (2017) found that 6–16-year-olds with ASD exposed to a second language experienced reduced clinical impact in executive function abilities as assessed by the parent informant-report Behavior Rating Inventory of Executive Function (BRIEF; Gioia et al., 2000) in a large sample of 174 children. In their study, there were no statistically significant differences between bilingual and monolingual children with ASD on parent-reported executive function outcomes; however, bilingually exposed children with ASD were less likely to have executive function ratings in the clinically significant range of concern. More recently, Ratto et al. (2020) found that young bilingual children with ASD (*n* = 55) were more likely to have significantly reduced parent-reported executive function difficulties on inhibitory self-control and flexible switching compared with monolingual children with ASD.

There is a growing but limited body of research examining the performance of bilingual children with ASD on directly assessed measures of executive function. A recent study investigating the relationship between bilingualism and executive function abilities in 6–9-year-old children with ASD with the dimensional change card sort task (DCCS) and the BRIEF parent report of executive functioning in daily life, found a tentative advantage in cognitive flexibility among 20 bilingual children with ASD compared with their monolingual peers (Gonzalez-Barrero & Nadig, 2019). Li and colleagues (2017) found a similar advantage for bilingual 8-year-old children with ASD using tasks assessing inhibitory control including the Stroop task (MacLeod & Grant, 1991), the Simon task (Simon, 1969), and the go/no-go task (Donders, 1969). More recently, Sharaan et al. (2021) investigated the impact of bilingualism on executive function domains relevant to the adaptive control hypothesis: sustained attention, inference control, and flexible switching utilizing the psychomotor vigilance task (Dinges & Powell, 1985), the Simon task, and the DCCS task respectively. The researchers found an advantage in sustained attention among bilingual children with ASD ages 5–12 compared with their monolingual peers with ASD (Sharaan et al., 2021). Together, these findings suggest that bilingualism does not negatively impact the executive function skills of children with ASD, and may even mitigate some difficulties in this domain. However, in each study a bilingual advantage was demonstrated in only one of the outcome variables assessed, and equivalence performance between bilingual and monolingual children with ASD was observed on all other executive functions. Therefore, it is still unclear whether these effects are robust and replicable.

## FUTURE DIRECTIONS

Future directions for this area of research include an increased attention to measurement issues. Laboratory-based measures of executive function tasks may have insufficient predictive value to actual participant ability in the real world (Crawford, 1998). For instance, a 30 ms difference in magnitude performance in the flanker task yields a significant inhibitory control advantage for bilingual children compared with their monolingual peers (Poarch, 2018); however, it is unclear whether this difference in executive function task performance constitutes an advantage in real life (Poarch & Krott, 2019). Well-documented executive dysfunction in ASD is not necessarily directly related to executive function deficits assessed experimentally (Geurts et al., 2009). Moreover, there is often poor convergence between neuropsychological and laboratory-based measures of executive function and more ecologically valid assessments of real-world behaviors (Dang et al., 2020). This is because laboratory-based behavioral measures index responses during structured situations, whereas informant-report measures probe how the individual functions in real-life situations. For this reason, it is imperative that future work include both standardized laboratory-based executive function measures and informant-based questionnaires that query executive functions in daily life (Uddin, 2021a).

Despite bilinguals and monolinguals being treated and compared as two uniform and distinct groups, it is rare that two bilinguals are the same (de Bruin, 2019). There is a broad range of bilingual profiles that is defined by individual experiences including age of acquisition, proficiency level, immersion in a bilingual environment, quantity and quality of switching between languages and so on. Given the absence of a single definition for bilingualism, it is possible to consider bilinguals with different degrees of language proficiency in different contexts such as school or home and different periods of their lives. Additionally, ASD is associated with a wide range of language abilities ranging from restricted to hyperlexic language abilities (Watson & Flippin, 2008). Nonetheless, current literature mainly reports only two profiles of ASD bilinguals: those raised in multilingual environments and those who know and can use several languages. As such, to date research on bilingualism in ASD does not reflect the diversity of language history profiles that is largely described in neurotypical populations (Digard et al., 2020). It is important that future work explore ASD language profile diversity, provide more information about the language context in which bilingual participants are immersed, and take into account the individual experiences of bilinguals. There are reliable assessments of multilingual profiles such as the Language Experience and Proficiency Questionnaire (Marian et al., 2007) available to provide a comprehensive description of bilingual participants. However, these types of nuanced measures of bilingualism are not yet routinely used in bilingualism research.

It is also important to note that not all children with ASD exhibit the same profile and severity of executive function deficits. This heterogeneity makes accurate assessment of ability all the more critical. Comorbidity with other conditions such as ADHD are very common among children with a primary diagnosis of ASD (Leitner, 2014). Executive dysfunction is more pervasive and severe in ADHD (Bloemen et al., 2018). Although not all children with ASD experience executive function difficulties (Baez et al., 2020), most children with comorbid ASD and ADHD exhibit executive dysfunction (Dajani et al., 2016). However, the nature and severity of executive dysfunction can greatly differ across and within children with ASD, ADHD, or comorbid ASD and ADHD (Uddin, 2021b). Future work must overcome the limited generalizability of small sample sizes and collect data from children of varied functioning levels with a range of comorbid conditions to better characterize relationships between bilingualism and executive function in ASD.

Future directions should explore brain mechanisms supporting putative bilingual executive function advantages in children with autism to better understand the neural circuitry underlying this phenomenon and understand how bilingualism differently affects the development of executive function systems in the brain of children with ASD. While a bilingual executive function advantage has been demonstrated in certain circumstances, the mechanisms that underlie this advantage remain elusive. Similarly, it remains unknown to what degree the development of a bilingual advantage for executive function is sensitive to IQ and age effects including acquisition age. For instance, a bilingual executive function advantage may only develop as a result of early exposure where both languages are used and activated in parallel for a long period of time. However, it may also be possible that the acquisition of a second language later in life is enough to influence executive control mechanisms. Future work should explicitly study the impact of IQ and developmental effects on the development of a bilingual advantage for executive function.

The ambiguous state of bilingualism research is not limited to executive function and extends to other aspects of mental functioning, such as theory of mind, which is commonly compromised in individuals with ASD (Rosello et al., 2020). Theory of mind is a socio-cognitive ability that is thought to be closely linked to executive functioning (Devine & Hughes, 2014). There are a few papers suggesting that bilingualism accelerates theory of mind development (Diaz & Farrar, 2018; Farhadian et al., 2010; Goetz, 2003; Han & Lee, 2013; Schroeder, 2018). Future work should continue to explore other cognitive domains in addition to executive function that bilingualism may influence.

## CLINICAL SIGNIFICANCE

Qualitative research on bilingual children with ASD demonstrates a discrepancy between evidence and instruction given to parents (Bird et al., 2012; Core & Hoff, 2015; Howard et al., 2021). Despite the fact the bilingualism does not have a negative impact on language development for children with ASD and may potentially compensate for executive dysfunction, practitioners working in the area of communication and language disorders continue to advise against providing children with developmental disabilities a bilingual environment (Baker, 2013; Bird et al., 2012; Drysdale et al., 2015; Jegatheesan, 2011; Yu, 2013, 2016a, 2016b). This may be because bilingualism is still often perceived as entailing a heavy cognitive load (Park, 2014).

After ASD diagnosis, bilingual children experience a rapid reduction in native language input, even though the use of the home language is persistent in adult-adult and adult-sibling interactions. The decision to restrict their bilingual child’s learning environment may have significant implications (Howard et al., 2020). For many immigrant families, the use of language in the home ties to cultural identity and facilitates communication and connectedness with their child. Language is an element of a community’s foundation and is inherently related to social development, thus, children being denied bilingual input often miss opportunities for social learning. Parents using their native language with their child better convey emotions, maintain engagement, and expand on topics (Wharton, 2000). However, when parents use their non-preferred language during family interactions, they have shortened interactions and more frequent interruptions (Yu, 2016a). Additionally, previous research indicates that bilingual parents report that limited second-language proficiency may influence their ability to interact fluidly with their child (Kremer-Sadlik, 2005). In the absence of evidence that bilingualism is detrimental to children with ASD, advising parents to restrict their bilingual language environment, thus altering the quality of interactions, may be problematic.

## CONCLUSIONS

The limited research available presents predominantly converging results on the relationship between bilingualism and executive function advantages in ASD. Developmental outcomes of bilingual and monolingual children on the autism spectrum are comparable. Recent large-sample behavioral studies of neurotypical adults and typically developing children do not support the bilingual advantage hypothesis. However, bilingualism may provide cognitive benefits in specific cases. For example, there is strong evidence that bilingualism acts as a protective factor and may buffer against cognitive decline and dementia. Likewise, bilingualism can bolster executive function in young children, particularly of low SES. And finally, there is preliminary evidence suggesting that bilingualism may ameliorate executive function difficulties in some children with ASD. This research in cognitive development and aging thus suggests that bilingualism might confer an advantage when executive function abilities are still developing, or in clinical populations where executive function is compromised. Thus, bilingualism, under the right conditions, may act as a protective factor for certain executive function difficulties.

The current review identified several topics that need to be addressed in future research. Firstly, the use of standardized objective proficiency measurements is strongly recommended. Measurement issues complicate the assessment of executive function difficulties and their neural basis as different combinations of laboratory-based measures, neuropsychological tests, and informant-reports have been used across studies. Future directions should include addressing these issues of measurement in order to maximize ecological and construct validity in research on the relationship of executive function and bilingualism. Secondly, detailed descriptions of the bilingual participants are necessary and individual differences should be better accounted for. Further work is needed to explore heterogeneity and comorbidity in order to better characterize relationships between bilingualism, executive function, and its neural representation in ASD.

## AUTHOR CONTRIBUTIONS

**Celia Romero**: Conceptualization: Equal; Writing – original draft: Lead; Writing – review & editing: Equal. **Lucina Q. Uddin**: Conceptualization: Equal; Visualization: Equal; Writing – original draft: Supporting; Writing – review & editing: Equal.
